# Predictors of Repeated PSA Testing Among Black and White Men From the Maryland Cancer Survey, 2006

**Published:** 2011-08-15

**Authors:** Yue Zhu, John D. Sorkin, Diane Dwyer, Carmela Groves, Eileen K. Steinberger

**Affiliations:** University of Maryland School of Medicine, Baltimore, Maryland; Baltimore Veterans Affairs Medical Center, Baltimore, Maryland; Maryland Department of Health and Mental Hygiene, Baltimore, Maryland; Maryland Department of Health and Mental Hygiene, Baltimore, Maryland; University of Maryland School of Medicine, Baltimore. Dr Steinberger is also affiliated with the Maryland Department of Health and Mental Hygiene, Baltimore, Maryland

## Abstract

**Introduction:**

Blacks have the highest incidence of and death from prostate cancer in the United States. Screening with prostate-specific antigen (PSA) may decrease mortality. Repeated testing allows for the calculation of PSA velocity (change of PSA over time), which may be a more clinically useful test for prostate cancer than a single PSA measurement. The objective of this study was to examine whether blacks were as likely as whites to report having had repeated PSA testing.

**Methods:**

The Maryland Cancer Survey 2006 was a population-based, random-digit−dialed statewide survey on cancer screening and risk behaviors of adults aged 40 years or older. We analyzed self-reported information on repeated PSA testing (2 PSA tests in the preceding 3 years) for 1,721 black and white men. We used logistic regression to estimate the effect of race and age on repeated PSA testing, adjusting for other covariates.

**Results:**

Sixty-five percent of men reported ever having had a PSA test; 41% had repeated PSA testing in the past 3 years. Blacks aged 40 to 49 were more likely to report having repeated PSA testing than whites in this age group (adjusted odds ratio [AOR], 3.3; 95% confidence interval [CI], 1.6-6.5). Blacks aged 60 to 69 were less likely to report repeated PSA testing than whites (AOR, 0.4, 95% CI, 0.2-0.8). No difference was seen by race among men aged 50 to 59 and men aged 70 or older. Repeated PSA testing was associated with living in an urban area and with having higher education, health insurance, a family history of prostate cancer, and having discussed cancer screening with a doctor.

**Conclusions:**

Self-reported repeated PSA testing differed by age and race, being higher among blacks aged 40 to 49 and lower among blacks aged 60 to 69, compared with whites in their respective age groups.

## Introduction

Excluding skin cancer, prostate cancer is the most frequently diagnosed cancer in men in the United States and is the second leading cause of cancer death after lung cancer (1). In 2008, there were an estimated 186,000 new cases of prostate cancer and more than 28,000 deaths. From 2000 through 2004, the age-adjusted incidence was approximately 60% higher among black men compared with white men (256/100,000 vs 161/100,000). The age-adjusted death rate was approximately 140% higher in blacks than whites for this same period (62/100,000 vs 26/100,000) (2).

Two screening tests are commonly used to detect prostate cancer: digital rectal examination (DRE) and serum prostate-specific antigen (PSA). In 2006, recommendations from the American Cancer Society (ACS) suggested that prostate cancer screening with PSA and DRE be offered annually to men aged 50 and older who have a life expectancy of at least 10 years. Men at high risk (including blacks and men with a family history of prostate cancer in 1 or more first-degree relatives diagnosed at an early age) should begin annual testing at age 45. Benefits and limitations of screening should be discussed so an informed decision about testing can be made (3). Interpretation of PSA concentration is difficult; there is no well-defined cutoff value for the diagnosis of cancer because of overlap in serum PSA levels in patients with benign disease and cancer (4). PSA level can increase with age and may be elevated in nonmalignant conditions such as benign prostatic hyperplasia and prostatitis (5). Prostate cancer can be present even at low PSA concentrations (≤3 ng/mL) (6,7).

Two large clinical trials have reported conflicting results on the effectiveness of PSA testing in decreasing death. The European Randomized Study of Screening for Prostate Cancer found that PSA screening reduced the rate of death from prostate cancer by 20% (8). The Prostate, Lung, Colorectal, and Ovarian Cancer Screening Trial in the United States found no significant difference in death rates from prostate cancer in men who were screened with PSA and men who were not (9).

Previous studies have shown the prevalence of PSA testing differs for whites and blacks. Gilligan et al examined Medicare and Medicaid claims data in New Jersey and concluded that elderly blacks were less likely to undergo PSA testing than were elderly whites (10). Fowke et al found differences in screening rates by race and age in a large sample of men attending community health centers in the southern United States; blacks younger than 50 were more likely to have had a recent PSA test than whites of the same age group, but among men older than 65, blacks were less likely to have had a recent PSA test (11). When examining a sample of men aged 40 to 49, Scales et al found that a higher proportion of black non-Hispanic men reported having had a PSA test than white non-Hispanic men (12).

PSA velocity is defined as the rate of change of serum PSA over time. Observational studies suggest the PSA velocity may be a more clinically useful screening test for prostate cancer than a single PSA concentration (13-15). A high PSA velocity may be a marker of high-risk prostate cancer or of prostate cancer aggressiveness (16,17). In 2007, the National Comprehensive Cancer Network guidelines for prostate cancer detection suggested that men with a PSA velocity higher than 0.35 ng/mL/y should consider biopsy, even if their PSA concentration is low (18). To date, however, no randomized trials have been conducted to determine the clinical usefulness of PSA velocity for decreasing cancer deaths.

Although several studies have examined the effect of race on prevalence of self-reported PSA testing, few have examined the effect of race on repeated PSA testing. The objective of our study was to determine whether black men were as likely as whites to report having had 2 PSA tests within the preceding 3 years in Maryland.

## Methods

### Research design

We performed a secondary analysis of data collected from the 2006 Maryland Cancer Survey (MCS), a population-based, cross-sectional statewide telephone survey on cancer screening rates, behavioral risk factors related to cancer, and access to health care among adults aged 40 or older living in Maryland (19) (Appendix). The random-digit–dialed survey employed disproportionate stratified sampling. Maryland was divided into 2 geographic strata: urban (consisting of Baltimore City and the 7 counties in the Metropolitan Baltimore-Washington, DC, area) and rural (consisting of the remaining 16 counties in western and southern Maryland and the Eastern Shore of Maryland). Genesys Marketing Systems Group (Fort Washington, Pennsylvania) provided a pool of 70,020 random telephone numbers. The rural area was oversampled, making up 40% of the telephone number pool, whereas the rural population represented 21.5% of the Maryland population.

REDA International, Inc (Wheaton, Maryland) conducted the MCS 2006 by using computer-assisted telephone interviewing technology. For each telephone number, 15 calling attempts were made. If someone answered the telephone, the number was confirmed to be a residential telephone number. (Cellular telephones and nonresidential numbers were excluded.) If REDA determined that at least 1 person aged 40 or older was living in the household, he or she was invited to participate in the survey. If 2 or more age-eligible people lived there, 1 was randomly selected to be interviewed. The anonymous survey lasted approximately 20 minutes. Respondents who spoke only Spanish were interviewed in Spanish by a bilingual interviewer.

A total of 61,273 telephone numbers were screened or called. Of these, 8.5% (5,187 telephone numbers) resulted in completed interviews. Of the numbers called, some (29.6%) were nonworking numbers; 13.6% were telephone numbers of a business or institution; and 4.3% were dedicated fax/modem numbers. Approximately 0.4% of the numbers were ineligible because of a language barrier (ie, a language other than English or Spanish was spoken). The remaining telephone numbers (43.6%) were ineligible for various reasons. The Council of American Survey Research Organizations response rate was 39.7%. The completion rate, defined as completed interviews/known eligible, was 75.1%.

People excluded from participation included those younger than 40, those who were unable to communicate because of a physical or mental impairment, and those living in group homes or institutions. The study was conducted between January and July 2006 and was approved by the institutional review boards at the University of Maryland, Baltimore, and the Maryland Department of Health and Mental Hygiene.

### Definition of variables

The study variable, race, was self-reported as white or black/African American. (Because of the small number of Hispanics who responded to the survey (n = 117), Hispanic ethnicity was not included as a separate race/ethnicity category.) The outcome variable, repeated PSA testing, was defined as a report of having had 2 PSA tests in the preceding 3 years. After a description of the PSA test was read, the following questions were asked: “Have you ever had a PSA test?” “How long has it been since you had your last PSA test?” and “How long before that PSA test was the previous one?” Respondents who reported having had 2 PSA tests within the previous 3 years were considered to have repeated PSA testing. Respondents who had never been tested, who reported having had only 1 PSA test, or who had had 2 PSA tests within a time frame longer than 3 years or within an unknown time interval were considered not to have had recent repeated testing. The first 2 questions are derived from the Behavioral Risk Factor Surveillance System Survey (www.cdc.gov/brfss/questionnaires/english.htm), and the third is from the 2003 Health Information National Trends Survey (hints.cancer.gov/questions/section1.jsp?section=Prostate+Cancer).

We included the following covariates in the analysis: area of residence (urban or rural), age in years (40-49, 50-59, 60-69, ≥70), education (high school graduate or less compared with some college or more), health status (excellent, very good, or good compared with fair or poor), having health insurance, having a family history of prostate cancer, and having a discussion about prostate cancer screening with a health care professional.

A total of 5,187 people were interviewed for the survey. We excluded people who did not report race (n = 38), women (n = 3,235), men whose race was not white or black (n = 72), and men who did not respond to the PSA question (n = 121), leaving 1,721 respondents for analysis.

### Statistical methods

Frequencies by race and repeat PSA tests were determined for all covariates; the χ^2^ statistic was used to evaluate the association between the outcome variable and covariates. The following characteristics were included in the logistic regression model: race, age, area of residence, education, health insurance, family history of prostate cancer, and discussion of prostate cancer with a health care professional. Data were analyzed with SAS version 9.1 (SAS Institute, Inc, Cary, North Carolina).

## Results

Compared with black respondents, whites were significantly more likely to report a high educational level, having some type of health insurance, and having discussed prostate cancer screening with a health care professional (Table 1). There was no difference in reporting a family history of prostate cancer by race. A higher proportion of white respondents reported having had repeated testing in the preceding 3 years. The unadjusted odds of repeated PSA testing were significantly higher in whites compared with blacks (unadjusted odds ratio [OR], 1.5; 95% confidence interval [CI], 1.1-2.0; *P* value = .01; data not shown).

The reported prevalence of repeated PSA testing varied by race and age (Table 2). Within race, repeated testing increased with advancing age among both blacks and whites. The prevalence of repeated testing by race varied by age group. A higher proportion of blacks than whites aged 40 to 49 reported repeated PSA testing; this was reversed in the group aged 60 to 69, in which fewer blacks than whites reported repeated PSA testing. The association between race and repeated PSA testing differed by age. After adjusting for other covariates, black men aged 40 to 49 had more than 3 times higher odds than white men of reporting repeated PSA testing. Blacks aged 60 to 69 had lower odds of reporting repeated PSA testing than whites (adjusted OR, 0.4; 95% CI, 0.2-0.8). Black men aged 70 or older were less likely to report repeated testing than white men, but the difference was not significant. No difference was seen by race for men aged 50 to 59.

We analyzed the adjusted association between repeated PSA testing and age, race, and other variables (Table 3). Comparing each race and age category to white men aged 50 to 59 as the reference, white men aged 40 to 49 had the lowest odds of reporting repeated PSA testing, followed by black men in that age group. There was no difference between the reference group and black men aged 50 to 59 and 60 to 69. Increased odds of repeated PSA testing were seen for white men aged 60 to 69 and 70 or older. Compared with the reference group, black men aged 70 or older had increased odds of repeated PSA testing but the finding was not significant. The odds ratios for the oldest white men (aged 60-69 and 70 or older) were higher than for black men of the same age groups. The odds of repeated PSA testing were higher among urban respondents, men with more than a high school education, men who had health insurance, and men who reported a family history of prostate cancer. The odds of having repeated PSA testing were highest in men who reported having discussed prostate cancer screening with a health care professional.

## Discussion

Our study found that repeated PSA testing differed by race and by age group. In the youngest age group (40-49 y), blacks were more likely to report repeated PSA testing than whites, whereas among men 60 to 69, blacks were less likely to report repeated PSA testing than whites. Whites aged 60 to 69 and 70 or older had the highest odds of repeat PSA testing.

Two studies were found examining the effect of race on repeated PSA testing. Mariotto et al used information from 2 data sources (2000 National Health Interview Survey and Medicare claims data) to model the distribution of age at initial PSA testing and the distribution of inter-screening intervals to generate individual PSA screening histories that reflected the population screening (20). Their modeling arrived at results similar to those of our analysis; blacks in younger age groups and whites in older age groups were more likely to have had repeated PSA testing.

Ross et al used data from the 2000 National Health Interview Survey to examine responses to the question "How many PSA tests have you had in the last 5 years?" (21). In the adjusted analysis, there was no difference by race among men 50 or older. Although the number of men aged 40 to 49 was small, a somewhat higher proportion of blacks than whites reported having had 3 tests in the last 5 years. As with our study, higher prevalence of repeated PSA testing was seen with higher educational levels and having health insurance. Although the question that defined repeated PSA testing was different from that used in our survey, the results were similar; younger blacks reported a higher rate of repeated PSA testing than whites. Our analysis differed from that by Ross et al in that we examined men aged 50 or older in 3 age groups and had differing results for race by age as noted above. Although the finding was not significant, blacks aged 70 or older also reported lower prevalence of repeated PSA testing. Prostate cancer screening is covered under Medicare Part B insurance. Men with only Medicare Part A (hospital insurance) may respond that they have health insurance yet not have the test because of lack of insurance that covers PSA testing; these questions were not asked during our survey.

At the time of the survey in 2006, ACS recommended that black men be offered prostate cancer screening earlier than white men, beginning at age 45, because they are at increased risk to develop prostate cancer compared with whites. This may explain why black men aged 40 to 49 were more likely to report repeated PSA testing. As has been shown previously, having a family history of prostate cancer yielded higher odds of reporting repeated PSA testing. Our analysis also showed that having a discussion about prostate cancer testing with a health care professional resulted in the highest odds of repeated PSA testing. This could be the result of recall bias: respondents who had discussed the test with their doctor better remembered having had the test. Alternatively, men who discuss the risks and benefits of prostate cancer screening may choose to be screened, believing that any test for cancer is better than no test at all.

As noted earlier, screening for prostate cancer with PSA is controversial. There is evidence that many of the cancers detected by PSA would not have otherwise been found, resulting in overdiagnosis (the diagnosis of cancer through screening that would not have been detected during the patient's lifetime) (22) and aggressive treatment of low-risk tumors (23). Today men with cancers at low risk for progression (Gleason score ≤6 and a clinical stage of T1c or T2a) are being offered a new treatment option, active surveillance, in which PSA tests and DREs are done more frequently (every 3-6 months) with prostate biopsies every 12 to 24 months. The goal of this strategy is to spare men with low-risk cancers the possible side effects of aggressive treatment, including incontinence and sexual dysfunction (24).

Our study is subject to limitations that could influence the results. The sample excluded people who do not have landline telephones. If men without landline telephones (cellular telephones only or no telephone) are, for example, less likely to have health insurance or routine screening, then our survey may overestimate the prevalence of prostate cancer testing. However, because the proportion of adults living in households with only cellular telephones decreases with increasing age, noncoverage bias based on telephone access probably introduces minimal bias into our study (25). The PSA test is a blood test and may be performed with other blood tests without the respondent's knowledge, which may underestimate PSA screening rates. All data in the 2006 MCS are based on self-report, and reports were not validated by comparison to medical records. Self-report could lead to an underestimate or overestimate of screening prevalence. We did not ask the reason why the initial or repeated PSA test was performed. Although most tests are done for cancer screening, the PSA test could also be drawn as follow-up to an already elevated PSA level, or after treatment for prostate cancer.

The study also has several strengths. The data came from a large statewide sample, and we were able to adjust for several covariates. This survey was offered in Spanish to those who preferred to respond in that language to reach a larger portion of the Latino community. Our results showing differences in PSA screening may be generalizable to other areas of the country because our sample was population-based.

Guidelines for prostate cancer screening have changed over time and vary by professional organization. Previously, the ACS recommended that PSA and DRE be offered annually to men who had a life expectancy of at least 10 years in a process of informed decision making (26). Guidelines stated that, if asked to make the decision for a patient, providers should have the patient tested. Currently, ACS recommends that men discuss the pros and cons of prostate cancer screening with their health care provider. For men who wish to be tested, ACS has guidelines for testing based on age, risk history, and results of PSA testing (27). The US Preventive Services Task Force statement, updated in 2008, recommends that health care providers discuss the "potential but uncertain benefits and the known harms" of prostate cancer screening and treatment with their patients before screening (28). The American Urological Association has developed a recent best practices statement (2009) that screening should be individualized following discussions between the health care provider and the patient (29). In addition, they recommend that the discussion and possible screening begin at age 40 for all men with a life expectancy of 10 years, to establish a baseline measurement with which future tests can be compared and PSA velocity can be determined. According to National Comprehensive Cancer Network guidelines, DRE and PSA testing are recommended for patients at age 40 to determine a baseline value. The time interval between follow-up tests should be based on the patient's initial PSA measurement, race, and family history of prostate cancer (30).

Although the guidelines for prostate cancer screening vary by organization, what is recommended by each is that men discuss the risks and benefits of screening with their health care providers and then make an informed decision before undertaking this testing. Once screening is begun, intervals for repeat screening should be based on PSA level, age, and family history. Our study found that a lower proportion of black men reported having had the discussion with their providers. We also found differences in repeated PSA testing by race and age, level of education, and health insurance status. If prostate cancer screening proves to be useful in the detection and management of prostate cancer, the disparities in screening need to be addressed and future studies need to be done to measure screening trends by race over time. In the meantime, the discussion about prostate cancer screening should become part of a routine medical visit for eligible men.

## Figures and Tables

**Table 1:** Baseline Characteristics of Male Respondents Aged 40 Years or Older Who Responded to Questions on Prostate Cancer Screening by Race, Maryland Cancer Survey, 2006

**Characteristic**	n (%)			***P* Value^a^**

	**Total (n = 1,721)**	**White (n = 1,497)**	**Black (n = 224)**	
**Area of residence^b^**				
Urban	987 (57.4)	806 (53.8)	181 (80.8)	<.001
Rural	734 (42.6)	691 (46.2)	43 (19.2)	
**Age, y**				
40-49	471 (27.4)	387 (25.9)	84 (37.5)	<.001
50-59	517 (30.0)	449 (30.0)	68 (30.4)	
60-69	348 (20.2)	310 (20.7)	38 (17.0)	
≥70	385 (22.4)	351 (23.4)	34 (15.2)	
**Education**				
High school graduate or less	554 (32.2)	462 (30.9)	92 (41.1)	.002
Some college or more	1,167 (67.8)	1,035 (69.1)	132 (58.9)	
**Health status^c^**				
Excellent to good	1,451 (84.8)	1,274 (85.5)	177 (80.1)	.04
Fair or poor	260 (15.2)	216 (14.5)	44 (19.9)	
**Has health insurance**				
Yes	1,626 (94.5)	1,421 (94.9)	205 (91.5)	.04
No	95 (5.5)	76 (5.1)	19 (8.5)	
**Family history of prostate cancer^c^**				
Yes	208 (12.3)	180 (12.2)	28 (12.8)	.80
No	1,486 (87.7)	1,295 (87.8)	191 (87.2)	
**Discussed prostate screening with a health care professional^c^**				
Yes	1,103 (65.0)	978 (66.2)	125 (56.8)	.006
No	594 (35.0)	499 (33.8)	95 (43.2)	
**Ever had PSA testing**				
Yes	1,115 (64.8)	982 (65.6)	133 (59.4)	.07
No	606 (35.2)	515 (34.4)	91 (40.6)	
**Had repeated PSA testing^d^**				
Yes	703 (40.9)	629 (42.0)	74 (33.0)	.01
No	1,018 (59.1)	868 (58.0)	150 (67.0)	

Abbreviation: PSA, prostate-specific antigen.

a Calculated by χ^2^ test.

b Urban defined as Baltimore City and the 7 counties in the Metropolitan Baltimore-Washington, DC, area; rural defined as the remaining 16 counties in western and southern Maryland and the eastern shore of Maryland.

c Some responses missing.

d Report of having had 2 PSA tests in the preceding 3 years.

**Table 2:** Adjusted^a^ Association of Repeated PSA Testing^b^ in Blacks Compared With Whites, Stratified by Age, Maryland Cancer Survey, 2006

Age, y	Blacks Who Reported Repeated PSA Testing		Whites Who Reported Repeated PSA Testing		AOR^a^ (95% CI)	*P* Value

	n (%)	*P* Value	n (%)	*P* Value		
40-49	17 (20.2)	.001	32 (8.3)	<.001	3.3 (1.6-6.5)	<.001
50-59	22 (32.4)		179 (39.9)		1.0 (0.5-1.8)	.98
60-69	16 (42.1)		192 (61.9)		0.4 (0.2-0.8)	.01
≥70	19 (55.9)		226 (64.4)		0.7 (0.3-1.5)	.32

Abbreviations: PSA, prostate-specific antigen; AOR, adjusted odds ratio; CI, confidence interval.

a Adjusted for area of residence, education level, health insurance, family history of prostate cancer, and having discussed prostate screening with a health care professional.

b Report of having had 2 PSA tests in the preceding 3 years.

**Table 3:** Adjusted Association Between Repeated PSA Testing^a^ and Different Predictors by Multiple Logistic Regression,^b^ 2006 Maryland Cancer Survey

**Characteristic**	Odds Ratio (95% CI)	*P* Value
**Race and age groups**		
**White**		
40-49 y	0.2 (0.1-0.3)	<.001
50-59 y	1 [Reference]	NA
60-69 y	2.7 (1.9-3.7)	<.001
≥70 y	3.0 (2.2-4.1)	<.001
**Black**		
40-49 y	0.6 (0.3-1.1)	.09
50-59 y	1.0 (0.6-1.9)	.98
60-69 y	1.0 (0.5-2.0)	.99
≥70 y	2.0 (0.9-4.4)	.09
**Area of residence**		
Urban	1.3 (1.0-1.6)	.04
Rural	1 [Reference]	
**Education**		
Some college or more	1.7 (1.3-2.2)	<.001
High school graduate or less	1 [Reference]	
**Health insurance**		
Yes	3.9 (1.6-9.7)	.003
No	1 [Reference]	
**Family history of prostate cancer**		
Yes	1.6 (1.1-2.3)	.01
No	1 [Reference]	
**Discussed prostate cancer screening with a health care professional**		
Yes	4.7 (3.5-6.2)	<.001
No	1 [Reference]	

Abbreviations: PSA, prostate-specific antigen; CI, confidence interval; NA, not applicable.

a Report of having had 2 PSA tests in the preceding 3 years.

b Adjusted for area of residence, education level, health insurance, family history of prostate cancer, and having discussed prostate screening with a health care professional.

## Appendix. Questions on Prostate Cancer Screening From the Maryland Cancer Survey, 2006

A prostate-specific antigen test, also called a PSA test, is a blood test used to check men for prostate cancer. Have you ever heard of this test?

1 YES
2 NO
7 DON'T KNOW/NOT SURE
8 REFUSED
9 NA

Have you ever had a PSA test?

1 YES
2 NO
7 DON'T KNOW/NOT SURE
8 REFUSED
9 NA

How long has it been since you had your last PSA test?

(READ ONLY IF NECESSARY)

1 Within the past year (<12 MONTHS AGO)
2 Within the past 2 years (≥1 YEAR BUT <2 YEARS AGO)
3 Within the past 3 years (≥2 YEARS BUT <3 YEARS AGO)
4 Within the past 5 years (≥3 YEARS BUT <5 YEARS AGO)
5 5 or more years ago
6 Never
7 DON'T KNOW/NOT SURE
8 REFUSED
9 NA

You said your last PSA test was [INSERT TIME FRAME]. How long before that PSA test was the previous one?

1 A year or less before
2 More than 1 but not more than 2 years before
3 More than 2 but not more than 5 years before
4 Over 5 years before
5 None before the most recent
7 DON'T KNOW/NOT SURE
8 REFUSED
9 NA
